# Diagnosing the undiagnosed: AI-enhanced multimodal modeling for placental mesenchymal dysplasia in high-risk pregnancies

**DOI:** 10.1097/MS9.0000000000003780

**Published:** 2025-08-29

**Authors:** Unaiza Naimal Ali, Azka Hassan, Maliha Khalid, Muhammad Talha, Aminath Waafira

**Affiliations:** a Department of Medicine, Fatima Memorial Hospital College of Medicine & Dentistry, Lahore, Pakistan; b Department of Medicine, Jinnah Sindh Medical University, Karachi, Pakistan; c Department of Medicine, King Edward Medical University, Lahore, Pakistan; d Department of Medicine, School of Medicine, The Maldives National University, Malé, Maldives

**Keywords:** artificial intelligence, Bayesian optimization, multimodal modeling, placental mesenchymal dysplasia

## Abstract

Placental mesenchymal dysplasia (PMD) is a rare vascular placental disorder that mimics molar pregnancy but often coexists with a viable fetus, making its misdiagnosis potentially devastating. In high-risk pregnancies, artificial intelligence (AI)-enhanced multimodal modeling – incorporating imaging, genomics, proteomics, and clinical features – offers a transformative diagnostic strategy. Leveraging Bayesian hyperparameter optimization for model refinement, this approach improves diagnostic accuracy while reducing uncertainty and clinician hesitation. Recent clinical studies support its efficacy and interpretability through SHAP and LIME models, while real-time surgical enhancements using Bayesian methods highlight its broader clinical utility. Despite current challenges such as data heterogeneity and integration barriers, multimodal AI provides unprecedented resolution in placental analysis, enabling precise differentiation between PMD and similar fetopathies. Ultimately, this advancement supports timely, non-invasive diagnosis, personalized management, and emotionally informed decision-making aligned with ethical AI implementation standards.

*To the Editor,*

Artificial intelligence (AI) in predictive modeling is revolutionizing early diagnosis and risk prediction in healthcare. It is especially helpful in detecting uncommon conditions such as placental mesenchymal dysplasia (PMD), a vascular placental anomaly that can mimic molar pregnancy but often occurs alongside a normal fetus. Misdiagnosis can lead to adverse fetal outcomes, particularly in high-risk pregnancies. AI algorithms like Bayesian hyperparameter optimization enhance model accuracy through optimal parameter selection using a surrogate model. Compared to random and grid search methods, Bayesian hyperparameter optimization has shown more consistent and stable results^[1]^.

Our computational strategy for prenatal diagnostics leverages multimodal AI combined with Bayesian hyperparameter optimization to address the limitations of conventional single-modality approaches. Clinical studies link PMD with fetal growth restriction in approximately 50% of cases^[2]^. An interesting case is that, in Kerala, a 23-year-old woman at 34 weeks was initially misdiagnosed via ultrasound with a partial molar pregnancy. However, placental histology later confirmed PMD, illustrating the risks of relying on imaging alone^[3]^. By integrating imaging, genomics, proteomics, clinical data, and computational modeling refined through hyperparameter optimization, we achieve a more accurate and individualized understanding of placental pathology. This enhances diagnostic pipelines, outcomes, and supports emotionally informed decisions for parents.

Recent clinical studies also show that AI models like SHAP and LIME are contributing to diagnostic pipelines by enhancing interpretability, which in turn fosters clinician confidence. Moreover, Bayesian optimization has demonstrated substantial improvements in real-time surgical applications, enhancing tissue recognition quality by 7.6% and achieving 99.5% user validation with reduced latency to 180 milliseconds^[4]^. These findings suggest that integrating multimodal AI is not just a research milestone but a viable clinical advancement. By integrating structural imaging, functional assessment, molecular profiling, and advanced computational modeling, researchers can now explore the placenta with unprecedented resolution, depth, and contextual understanding^[5]^.

Even with all the progress, multimodal AI in healthcare still comes with challenges like data heterogeneity, the absence of integrated datasets, and the complexity of merging inputs from imaging, clinical notes, and genomics. These are all compounded by interpretability concerns, population biases, and the challenge of bringing such systems into healthcare settings, especially in low-resource environments that may lack the infrastructure needed to support the deployment of these advanced technologies^[4]^. Yet as ongoing research continues to develop these technologies further, their use in routine obstetric practice has the potential to transform the norm of prenatal diagnosis into one of early, non-invasive, and psychologically guiding interventions.

This convergence of streams of data is not merely methodologically innovative but clinically essential. Proper and timely diagnosis of PMD avoids the common misdiagnoses that result in the termination of viable pregnancies unnecessarily. Multimodal strategies provide in real-time visualization of the placenta as a living, dynamic organ that is capable of responding to environmental influences. AI-driven diagnostics provide unambiguous differentiation between PMD, molar pregnancy, and other fetopathies, aiding proper NIPT, timely amniocentesis, and better perinatal management. This strategy finally provides clinical accuracy while aiding the emotional readiness of expectant parents. Our work is in line with the TITAN Guidelines on the need for transparency in AI use in healthcare^[6]^.

## Data Availability

No data were generated for this manuscript.

## Research registration unique identifying number (UIN)

1. Name of the registry: N/A.

2. Unique Identifying number or registration ID: N/A.

3. Hyperlink to your specific registration (must be publicly accessible and will be checked): N/A.

## Peer and provenance statement

This article was not commissioned and was externally peer-reviewed.
